# Preparation and Performances of Warp-Knitted Hernia Repair Mesh Fabricated with Chitosan Fiber

**DOI:** 10.3390/polym11040595

**Published:** 2019-04-01

**Authors:** Shuang Yu, Pibo Ma, Honglian Cong, Gaoming Jiang

**Affiliations:** 1 Engineering Research Center for Knitting Technology, Ministry of Education, Jiangnan University, Wuxi 214122, China; yushuang_ysh@163.com (S.Y.); jgm@jiangnan.edu.cn (G.J.); 2 State Key Laboratory of Bio-Fibers and Eco-Textiles, Qingdao University, Qingdao 266071, China

**Keywords:** hernia repair mesh, chitosan fiber, warp-knitted mesh, mechanical properties, antibacterial property

## Abstract

In this paper, warp-knitted knitted fabrics with chitosan fibers for ventral hernia repair were fabricated with three kinds of structures. The properties of chitosan fiber, yarns, and fabrics were tested. The results demonstrated that the properties of a mesh fabricated with 1-0/1-2/2-3/2-1// structure were slightly better than those of other fabrics. The mechanical properties of the three produced fabrics were weak. However, the results demonstrated that chitosan meshes have many advantages, such as excellent hygroscopicity, and thermal and antimicrobial properties, which makes them one of the best materials for ventral hernia repair. The findings have theoretical and practical significance for the industrial uses of chitosan in ventral hernia repair.

## 1. Introduction

Hernia is one of the common surgical diseases. Hernia mesh repair is the use of screen-like surgical implants to repair hernias. Currently, tension-free hernia repair and laparoscopic hernia repair are common ways used in ventral hernia repair. The prosthetic mesh applied plays an important role in ventral hernia repair, which determines the success rate, recurrence of operation, and postoperative recovery degree. Nowadays, common hernia repair meshes have good physical and mechanical properties [1]. Examples include polypropylene mesh (PPM) [2,3], polyvinylidene fluoride (PVDF) mesh [4,5,6], polytetrafluoroethylene (PTFE) mesh, expanded polytetrafluoroethylene (ePTFE) mesh, and polyester (POL) mesh [7], but they are all non-absorbable materials and lack good biological characteristics [8]. Hence the absorbable mesh is becoming a hot research field of ventral hernia repair at present.

Chitosan is one of the best materials for ventral hernia repair because of its many advantages, such as non-toxic, non-irritating, good hygroscopicity, good antimicrobial property, biocompatibility, and degradability [9,10]. Starting as early as the 1980s, studies on the application of chitosan in artificial skin have made great progress. Scientists developed artificial skin made by cross-linking chitosan with collagen, which greatly improved performance capabilities [11]. Other related studies also demonstrated through experiments that cell proliferation and infiltration of the artificial skin scaffolds made by coating chitosan on the collagen skeleton were significantly improved, confirming the good biological compatibility of chitosan [12]. Scientists also found that it was difficult to reconstruct and repair bone tissue after injury due to certain mechanical stress, antimicrobial property, and biocompatibility requirements of materials [13]. Pangon discovered that the application of chitin crystals, chitosan, and hydroxyapatite can improve the mechanical properties of bone tissue reconstruction and reduce the recovery time due to the good antimicrobial and biocompatibility properties of chitosan. In the study [14], the knitted fabric used is flexible, open, and has a microporous effect.

Some scholars found that polypropylene mesh (PPM) knitted by warp-knitting technology did not disturb the normal anatomy and had no suture tension. PPM has high porosity, sufficient strength, good biocompatibility, low infection rate, excellent fixation performance, and host growth performance. The patients suffer less, and their activities do not need to be severely restricted. Besides, postoperative recurrence rate was low. For good mechanical properties, the mesh was designed with 18–20 courses per centimeter [15]. Mirjavan designed five warp-knitted fabrics which had different types of structures (Tricot, Pin-hole-net, quasi-Sandfly, Sandfly, and quasi-Marquissite) for healing hernia [16]. The study established that the quasi-Marquissite mesh was the best structure among these five fabrics. Lu developed a new composite mesh employing the two-bar warp-knitting technique; the study showed that porosity and structure of mesh had effects on mechanical properties [17]. Dumont focused on the processing and antibacterial properties of chitosan-coated alginate fibers [18]. Amato studied the antimicrobial activity of catechol-functionalized-chitosan versus *Staphylococcus epidermidis* [19].

At present, although chitosan has many applications in the biological field, there are few studies on its application for hernia repair mesh using warp-knitting technology with chitosan. Therefore, it is extremely imperative to use chitosan to produce a mesh widely used in hernia repair through high-speed warp-knitting equipment. In this paper, chitosan fiber has been selected for the production of three kinds of fabrics through warp-knitting technology. The properties of chitosan fiber, yarn, and fabrics are studied.

## 2. Experimental Setup

### 2.1. Material

Chitosan fibers with a degree of deacetylation of 86% were obtained from Jifa knitting factory, (Qingdao, Shandong, China). The antimicrobial property tests of the materials were provided by Guangzhou Fiber Product Testing Institute (Guangzhou, Guangdong, China). All aqueous solutions were prepared with ultrapure water. All samples were used as purchased without further purification.

### 2.2. Preparation of Chitosan Knitted Fabric

**Figure 1:**
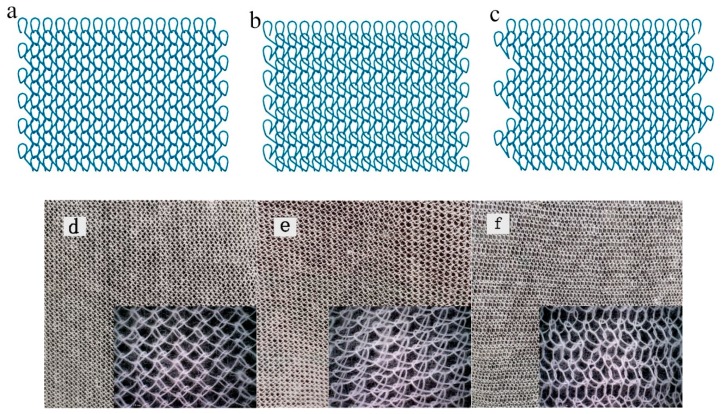
Structure of three fabrics: No. 1 (**a**,**d**), No. 2 (**b**,**e**), and No. 3 (**c**,**f**).

**Figure 2:**
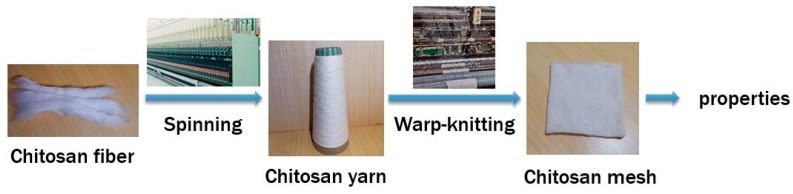
Preparation of chitosan knitted fabrics.

The structure of the mesh becomes particularly important based on the material used. In terms of dimensional stability, porosity, and especially suture performance, knitted fabrics are more flexible, open, and porous than braided ones. Generally, warp-knitted fabrics including both basic and complex structures are not easy to unravel. Different fabric structures also tend to impart great influence on physical properties of the mesh. The fabrics produced via single guide bars are strong, porous, stable, and light. The tricot is more flat, porous, and possesses good extensibility. At present, the atlas is used in domestic and foreign markets for tension-free hernia repair, which is relatively simple, can reduce the rigidity of single bar warp-knitted fabric, and increase the softness and adaptability of knitted fabric. The yarn of chitosan with a linear density of 14.6 tex was knitted using a Raschel warp-knitting machine (E14), (Aoyuan Textile Machinery Co. Ltd., Changzhou, Guangdong, China. Chitosan knitted fabrics for ventral hernia repair were fabricated with three kinds of structures shown in Figure 1, of which No. 1 is 1-0/1-2//, No. 2 is 1-0/2-1//, and No. 3 is 1-0/1-2/2-3/2-1//. Figure 2 illustrates the above process in a simplified sequence flow diagram.

### 2.3. Characterizations

Samples were tested under a temperature of 20 ± 2 °C and relative humidity of 65% ± 2%, which is a standard atmospheric environment, for 24 h.

X-ray diffraction (XRD) measurements were conducted on a D2 PHASER x-ray diffractometer, (Bruck AXS GMBH, Karlsruhe, Germany). Cu radiation generated at a voltage of 40 kV and current of 40 mA was utilized. The scanning range was varied from 5° to 50° at a rate of 4°/min.

Fourier transform infrared spectroscopy (FTIR) was performed on a Nicolet iS10 spectrometer (Semel Fisher Technology Co., Ltd., MA, USA by the KBr particle technique. The range of chitosan was varied from 5000 to 400 cm^−1^.

Thermogravimetry (TG) was tested by a Q500 Thermal Gravimetric Analyzer (TA Instruments, New Castle, DE, USA). The nitrogen flow rate was 40 mL/min, and the temperature range varied from 30 to 900 °C at a rate of 10 °C/min.

Yarn quality can be reflected by breaking strength and elongation at break. Polyester, polyamide, cotton, and chitosan yarns with the same linear density were compared by MTS Exceed E43 (MTS Systems (China) Co., Ltd., Guangzhou, Guangdong, China) according to the National Standard GB/T3916-1997. The speed was 500 mm/min. The gap between the upper and lower chucks was 500 mm and the pretension was 1 cN.

Yarn evenness refers to thick and thin irregularities along the yarn length direction. The unevenness of yarn has an effect on the processing and quality of the fabric to some extent. Based on the National Standard GB/T3292-1997, the evenness of yarn was checked on a YG135G evenness tester (Shaanxi Changling Co., Ltd., Baoji, Shaanxi, China). Twist is usually expressed by the number of twists within the length of the yarn in 10 cm. The commonly used indices are twist, twist direction, and twist unevenness. The twist direction is divided into S twist and Z twist. The yarn twist was measured through the twisting method by a Y331N yarn twist counter (Nantong Hongda Experimental Instrument Co., Ltd., Nantong, Jiangsu, China). The clamping length was 250 mm.

### 2.4. Mechanical Properties

The breaking strength test is used to ascertain the warp and weft strength of the fabric. The knitted fabric is deformed greatly due to the transfer of the loops in the stretching process, which often leads to obvious shrinkage in the non-stretching direction. The shear stress caused by the specimen at the clamp mouth is particularly concentrated, which causes the fracture of most strips near the clamp mouth and affects the accuracy of the experiment. In order to improve the situation, a trapezoidal test strip was used. According to the National Standard GB/T3923.1-1997, the breaking strength of three kinds of specimens was determined using an HD026N+ electronic fabric strength tester (Nantong Hongda Experimental Instrument Co., Ltd., Nantong, Jiangsu, China). Five samples of both weft and warp direction were produced. The clamping distance was 200 mm, and tensile speed was 100 m/min with 2 N of pretension.

Bursting strength is one of the key parameters used to evaluate the clinical applicability of the mesh. Bursting refers to the phenomenon in which the fabric expands and gradually destroys under the external force perpendicular to the fabric plane. The force mode of bursting strength is multi-directional damage. The mesh, when in use, is stressed by abdominal pressure due to different movements such as coughing, sitting, standing, and walking. The deterioration of bursting strength will lead to poor effect after implantation and high recurrence rate. The bursting strength of three kinds of specimens was also tested by the HD026N+ electronic fabric strength tester according to the National Standard GB/T19976-2005. There were five samples each per fabric with a size of 100 mm × 100 mm. The clamping distance was 350 mm with a speed of 100 m/min.

According to the National Standard GB/T 3917.2-1997, the tearing strength of three kinds of specimens (single rip) was tested by the HD026N+ electronic fabric strength tester. There were five samples of each fabric with a size of 200 mm × 50 mm. The clamping distance was 100 mm with a speed of 100 m/min.

### 2.5. Acid and Alkali Resistance

Solutions of pH = 1–14 were prepared with 38% hydrochloric acid, K_2_CO_3_, and ultrapure water, respectively. The samples of three different fabrics were cropped into sizes of 2 cm × 2 cm. The fabrics were simultaneously soaked, and the change of samples in the solutions was observed.

### 2.6. Antibacterial Property

Antimicrobial activity is an important property of chitosan. According to the National Standard GB/T 20944.2-2007 titled Evaluation of the Antibacterial Properties of Textiles Part II: Absorption method—the study group and the control group were inoculated with bacteria, followed by immediate elution and post-culture elution, respectively. The number of bacteria in the elution was determined, and the bacteriostasis value or bacteriostasis rate was calculated to evaluate the antibacterial effect of the samples. The bacteria used in this study were *Escherichia coli* and *Staphylococcus aureus*.

The specific steps were as follows: *E. coli* and *S. aureus* were amplified and diluted to 1.0 × 10^8^ cfu/mL each as the experimental solutions. A microbial inoculation of 750 μL was taken to the surface of the fabrics under the condition of 37 °C for 3 h. The fabrics inoculated with bacteria were transferred to the centrifuge tube for ultrasound for 10 min to elution the bacteria on the surface. After the eluent dilution, 10 μL bacteria liquid was evenly coated in an agar culture plate (Shanghai Jing An Biological Technology Co., Ltd., Shanghai, China). It was then turned upside down and cultivated for 24 h at 37 °C after medium solidification.

The antibacterial ratio (A) was calculated as follows, in which *C_t_* was the total number of the bacteria in the study group, and *C_o_* was the number of bacteria in the control group.

(1) $$A=\frac{C_{t}-C_{o}}{C_{t}}\times 100\%$$

## 3. Results and Discussion

### 3.1. Properties of Chitosan Fiber

#### 3.1.1. Basic Performances of Chitosan Fiber

Table 1 shows the basic performances of the chitosan fiber. Linear density determines the strength of the yarn and affects the strength of the fabric. Morphology, structure, and mechanical properties of fibers, yarns, and fabrics are affected by hygroscopic properties. The breaking strength and elongation of breaking are important indexes of mechanical properties of fiber, so fibers are required to have certain resistance to external forces in processing and use. Additionally, the crimp can increase the mechanical entanglement and binding force between fibers, which is conducive to the formation of mesh and strip in the process of spinning and the production efficiency of spinning.

#### 3.1.2. XRD Test and FTIR Test

**Figure 3:**
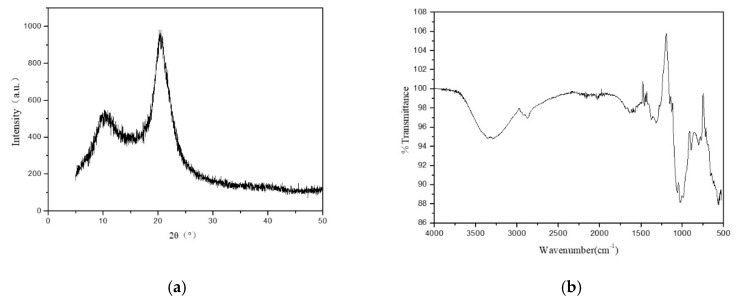
(**a**) XRD curve of chitosan fiber, and (**b**) infrared spectrum curve of chitosan fiber.

There are two obvious characteristic diffraction peaks at 2*θ* = 10.77° and 20.57° for chitosan, as shown in Figure 3a. The intensity of the diffraction peak is large and sharp, which indicates that the chitosan fiber has high crystallinity. There are crystallization zones and amorphous zones among the fiber macromolecular chains. The percentage of the crystallization area in the whole fiber is called the crystallinity of the fiber. Compared with the amorphous zone, the macromolecules have many strong connection points between them, which can bear large external forces and have small deformation and stable structure. The chitosan fiber has good physical and mechanical properties, accordingly. As shown in Figure 3b, the absorption band at 3355 cm^−1^ was assigned to –OH and –NH_2_, and the absorption band at 3290 cm^−1^ was the stretching vibration of –OH, too. The absorption band at 2868 cm^−1^ was the –CH stretching vibration. There are three small peaks of C–O stretching vibration at 1317, 1057, and 1024 cm^−1^.

#### 3.1.3. TG Test

**Figure 4:**
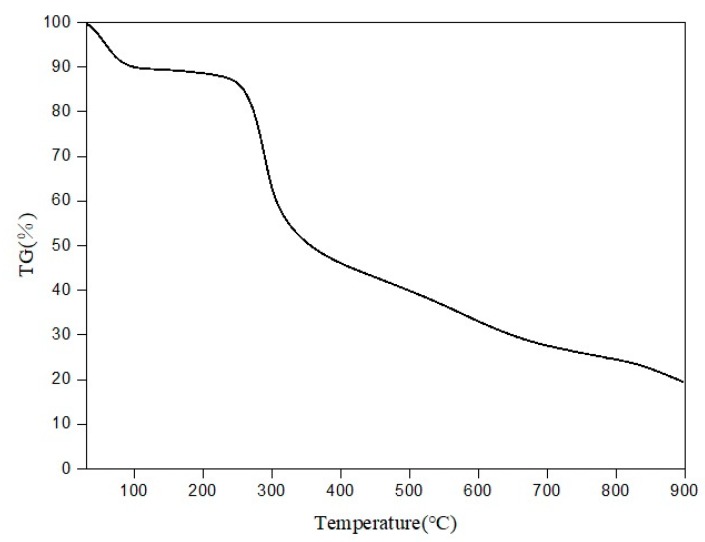
Thermogravimetry (TG) curve of chitosan fiber.

According to the TG curve shown in Figure 4, there were three stages in the process of thermal decomposition with an initial weight of 6.183 mg. It started to decompose at 260 °C. The first stage was not obvious and occurred at about 72 °C, in which the weight loss rate was up to 8.02%. The main reason was the loss of water in the fiber, which was related to the excellent moisture absorption of the chitosan. The second stage occurred at 260–315 °C with 57% of the initial weight, which continued up to 565 °C. The reason why the weight loss decreased sharply was the decomposition of the molecular chain of chitosan. The chain length became shorter, and the crystallinity of the system decreased due to the change of the crystalline region, that was why the weight decreased greatly. The third stage of weightlessness was at 565–900 °C, in which the mass loss rate was 15.93%. The fiber was degraded completely at 900 °C. The carbon residue rate was 19.63% with a weight of 0.596 mg. That was the formation of a dense carbon layer on the surface at high temperature, which prevents further oxidation and decomposition of the chitosan. The large amount of carbon residue in chitosan is responsible for its good flame retardancy. The highest temperature in the processing and use of the fabric is lower than 200 °C. The properties of chitosan did not change until the temperature was higher than 200 °C. Therefore, chitosan has good thermal stability.

### 3.2. Properties of Chitosan Yarn

#### 3.2.1. Strength Test

**Figure 5:**
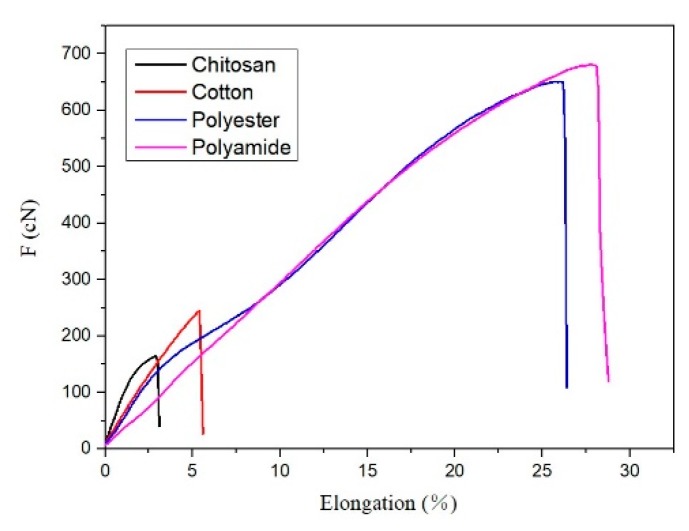
The breaking strength of four yarns.

Yarn will usually be subjected to tension and repeated load during preparation and knitting, which directly affects the properties of the resultant knitted fabrics. Strength of yarns needs to meet these production strength requirements. However, according to the data shown in Figure 5, the breaking strength and elongation at break of chitosan yarn were the lowest among the four yarns with the same linear density, and the tensile strength was only 164.4 cN. The mechanical properties of the chitosan yarn were quite poor.

#### 3.2.2. Evenness Test

The evenness of yarn is an important index of knitting yarn, which can be expressed by CV value, fine end, slub, nep, and so on. Fine end, slub, and nep are all yarn defects, which are different from normal yarn, or coarse or fine. The lower the CV value, the more uniform the yarn and the better the effect of the fabric. It is difficult to weave smoothly if the yarn has many slubs, resulting in yarn breakage, damage to the machine, and formation of “horizontal strip” and “cloud spot” on the appearance of the fabric easily. If there are many fine ends in the yarn, it causes more broken ends due to insufficient strength. Consequently, it will affect the quality of fabric and reduce productivity of the machine. Table 2 shows that the evenness of chitosan yarn is low and the quality is poor compared with cotton yarn with the same linear density, but the defects and impurities of yarn can be removed by the cleaning device of the winding machine.

#### 3.2.3. Twist Test

The twist of knitted yarn is lower than that of woven yarn. Excessive twist will affect loop spirality and result in defects of fabrics. It will also affect the softness and elasticity of the fabric. When the twist is too small, the yarn strength decreases, which leads to the increase of yarn breakage during the weaving process. At the same time, the yarn is expanded, and the pilling phenomenon is especially serious for fabrics. To increase strength and reduce breakage, the yarns of chitosan are more tightly twisted than the yarns of cotton with the same linear density owing to the low strength, thus increasing strength and reducing breakage. The usual twist number of cotton yarn with linear density of 14 tex is 88 ± 2 twists/10 cm. The twist direction of chitosan yarn with linear density of 14.6 tex is Z, the twist number is 183.04 ± 2 twists/10 cm, and the twist unevenness is 1.59%. It can be seen that the twist and twist unevenness of chitosan yarn meet the requirements of knitting.

### 3.3. Properties of Chitosan Mesh

#### 3.3.1. Basic Parameters of Chitosan Mesh

Table 3 shows the basic parameters of three kinds of chitosan knitted fabrics. The weight per unit area of fabrics not only affects wearing performance, but also serves as an important basis of economic calculation. Moreover, the smaller the fabric thickness, the more comfortable the patient will feel. These indicators are fundamental to the success of the operation and postoperative recovery [20]. All three kinds of fabrics belong to lightweight mesh with little difference in thickness. Lightweight mesh has many advantages [21,22,23]. Porosity reflects the size and pore distribution of the mesh, which is one of the important indexes to evaluate whether or not the mesh meets clinical specifications [24,25]. Appropriate porosity is conducive to postoperative recovery. Therefore, on the premise of ensuring sufficient strength and stiffness, the selection of appropriate porosity is also of great clinical significance for the development of mesh in hernia repair.

#### 3.3.2. Mechanical Properties

The main function of the mesh is to resist the internal pressure of the human body and support the new tissue. The stable structure and sufficient strength of the mesh need to meet the requirements. The quality of the mesh is evaluated by the main physical and mechanical properties, such as tensile strength, tearing strength, and breaking strength tests. The mechanical properties of chitosan knitted fabrics are shown in Table 4.

Because of the high elasticity of the knitted fabric, the chitosan fabric breaks at the jaw during tensile process. Overall, the breaking strength of chitosan fabrics is poor, both vertically and horizontally. For sample No. 2 and sample No. 1, the breaking strength and elongation at break in the weft direction were higher than those in the warp direction. Sample No. 3was in contrast to them. The strength of sample No. 3 was a little higher than that of the others in the warp direction, and the strength of sample No. 2 and sample No. 1 was slightly higher than that of the other two kinds of structures in the weft direction. The Table 4 shows the tensile properties of the three kinds of mesh.

Bursting strength is also one of the key indicators to evaluate whether the mesh can be used in clinical practice. The properties of resistance to repair hernia are increased with the bursting strength. The breaking strength of human abdominal fascia is about 232 N. However, the three kinds of mesh were not up to the standard of application. The deterioration of bursting strength will lead to poor effect after implantation and high recurrence rate. The longitudinal of three fabrics can be torn, but the transverse cannot be torn normally. The causes of this phenomenon are as follows: Firstly, the warp-knitted structure of the mesh determines the characterization of its tearing properties. In the process, the yarn is more easily destroyed than the loop in the longitudinal direction. The loop is not easily destroyed in the transverse direction; the direction is changed during tearing. Therefore, the longitudinal tearing strength of the three kinds of mesh was studied in this experiment. From Table 4, it can be seen that the tearing strength of sample No. 3 is greater than that of sample No. 2 and sample No. 1, which indicates that the longitudinal tearing strength of sample No. 3 is greater than that of the others in the effective area. The minimum peak value of the three samples was the same. The ability of sample No. 3 to resist tearing damage was better than that of the others.

#### 3.3.3. Acid and Alkali Resistance

**Figure 6:**
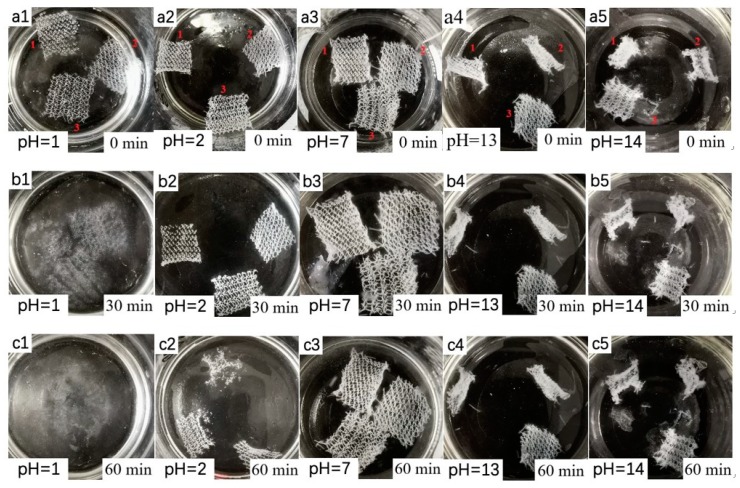
Images of three fabrics treated in (**a1**,**a2**) acid solution, (**a3**) ultrapure water, and (**a4**,**a5**) aqueous alkali at the beginning; three fabrics treated in (**b1**,**b2**) acid solution, (**b3**) ultrapure water, and (**b4**,**b5**) aqueous alkali after soaking for half an hour; three fabrics treated in (**c1**,**c2**) acid solution, (**c3**) ultrapure water, and (**c4**,**c5**) aqueous alkali after soaking for an hour.

It was found that in the hydrochloric acid solution of pH = 1, the three kinds of fabrics began to dissolve in eight minutes, but there was no change with regards to the other groups. After soaking the three kinds of fabrics in a solution with pH = 1 (b1) for half an hour, the samples disappeared as shown in Figure 6. The fabrics in a solution of pH = 2 (b2) began to be dissolved, and the tricot tended to be curled up in a solution of pH = 14 (b5), but this was not obvious with the atlas sample. After soaking for an hour, the fabric largely dissolved in a solution of pH = 2 (c2), and the three fabrics in a solution of pH = 13 (c4) also curled up. It was observed that fabrics of chitosan were difficult to dissolve in neutral and alkaline solutions and have high solubility only in strong acid solutions. At the same time, the fabric curled up in a strong alkaline solution.

**Figure 7:**
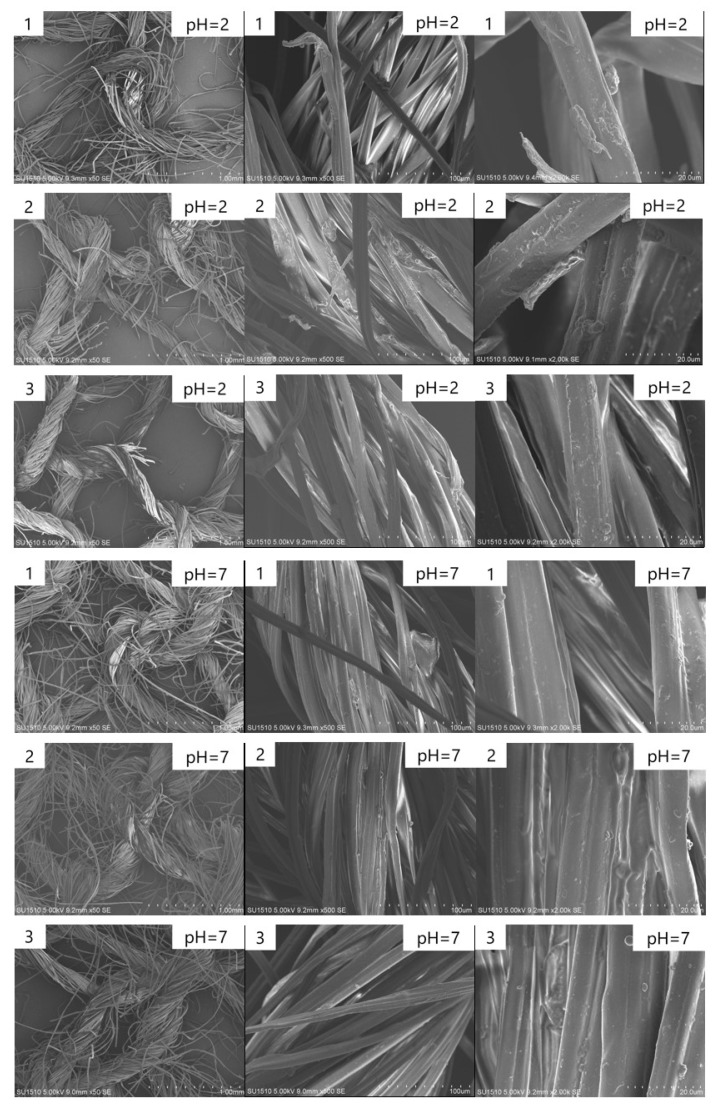

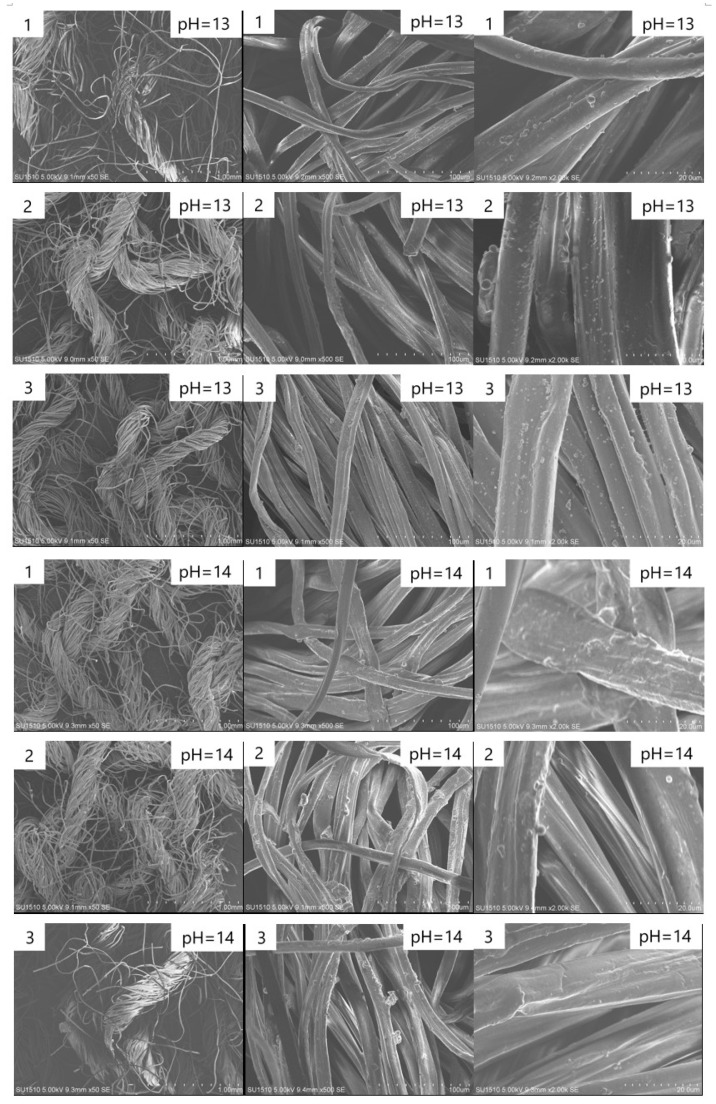
SEM images of three fabrics treated in acid solution, ultrapure water, and aqueous alkali for half an hour.

The microscopic morphologies of the three kinds of fabrics treated in the different solvents for half an hour were investigated, and the images are shown in Figure 7. The structure of the three fabrics treated in the hydrochloric acid solution of pH = 1 was not preserved, as most of them had been dissolved within half an hour, so electron microscopy experiments were not conducted on them. It was found that after half an hour of treatment with pH = 2 solution, the yarn would break, and the fiber surface would be damaged. When treated in an aqueous alkali of pH = 13 and pH = 14, yarn breakage was less than when treated in an acid solution of pH = 1; the yarn would break less than in the acidic solution, but damage would also occur on the fiber surface.

#### 3.3.4. Antibacterial Property

Chitosan has a good sensitivity to a variety of bacteria. In recent years, many studies have been done on the antimicrobial activity and mechanism of chitosan; however, the results were not consistent. Three possible antimicrobial mechanisms were summarized: Charge action, biological binding, and metal chelation. Charge action [26], however, appears to be most predominantly applied theory. Its mechanism is such that, the electrostatic interaction between positively charged chitosan molecules and negatively charged bacterial cell membranes causes change and damage of the bacterial surface membrane. This inhibits the metabolism of bacteria and results in the death of the bacteria [27]. There are many other factors that affect the antibacterial activity of chitosan [28,29], including molecular weight [30] and degree of deactivation [31]. The rate of bacterial inhibition against *E. coli* of the three kinds of fabric was 99.99%, 99.75%, and 99.60%, and the rate of bacterial inhibition against *S. aureus* of the three kinds of fabric was 99.99%, 99.97%, and 99.92%, respectively. According to the National Standard GB/T 20944.2-2007, the three kinds of pure chitosan fabrics had excellent antimicrobial effect against *E. coli* and *S. aureus*.

## 4. Conclusions

In this paper, the properties of chitosan fibers with a deacetylation degree of 85% and yarns spun using them were tested. The results showed that chitosan had good thermal and moisture absorption properties. Subsequently, three kinds of warp-knitted meshes were prepared with chitosan yarn of 14.6 tex. Among these fabrics, the properties of the sample No. 3 were slightly better than those of the other fabrics considering all merits and defects of these meshes prepared in this study. The warp-knitted mesh with chitosan fiber is difficult to be dissolved in neutral and alkaline solution and has high solubility only in strong acid solution. In addition, all kinds of fabrics had better antibacterial effect against *E. coli* and *S. aureus*, and the antibacterial rate was over 99%. So many factors have been found to affect the performance of meshes used in hernia repair. The authors opined that, should the mechanical properties of chitosan fabric be improved, it would be an ideal hernia repair material in the future.

## Figures and Tables

**Table 1:** Basic performances of chitosan fiber.

Appearance	Degree of Deacetylation/%	Ash/%	Linear Density/Dtex	Length/mm	Crimpness/mm	Moisture Regain/%	Breaking Strength/Cn	Elongation of Break/%
White	86%	0.94	1.6	38 ± 1	3.1	15.68 ± 0.03	3.18 ± 0.02	18.1 ± 0.09

**Table 2:** The evenness of chitosan yarn.

Parameters	U (%)	CVm (%)	Fine End (−50%)/km	Slub (50%)/km	Nep (200%)/km
Yarn of chitosan	16.79	21.85	2240.00	0.00	1320.00
Yarn of cotton	13.87	15.88	520.00	0.00	20.00

**Table 3:** Basic parameters of three kinds of fabrics.

No.	Weight per Unit Area (g/m^2^)	Density		Thickness (mm)	Porosity (%)
		Course	Wale		
1	39.42 ± 0.10	54 ± 0.00	22 ± 0.00	0.336 ± 0.00	29.59 ± 0.11
2	42.63 ± 0.09	26 ± 0.00	23 ± 0.00	0.332 ± 0.00	33.75 ± 0.06
3	42.70 ± 0.06	22 ± 0.00	28 ± 0.00	0.330 ± 0.00	37.43 ± 0.08

**Table 4:** Mechanical properties of three kinds of fabrics.

No.			1	2	3
Breaking strength	Breaking strength (N)	Warp	20.26 ± 1.78	17.36 ± 1.40	28.96 ± 1.46
		Weft	26.80 ± 1.44	21.54 ± 1.21	22.28 ± 1.71
	Elongation at break (%)	Warp	8.714 ± 1.070	10.360 ± 0.802	10.812 ± 0.970
		Weft	14.746 ± 0.846	14.786 ± 0.900	14.650 ± 0.721
Bursting strength	Bursting strength (N)		93.60 ± 4.74	71.00 ± 2.99	74.10 ± 5.76
	Bursting height (mm)		9.29 ± 0.95	11.80 ± 1.23	9.00 ± 0.10
	Bursting work (J)		0.043 ± 0.89 × 10^−4^	0.040 ± 0.00	0.023 ± 0.22 × 10^−4^
Tearing strength	Average peak value (N)		1.134 ± 0.01	1.475 ± 0.02	1.895 ± 0.01
	Maximum peak value (N)		2.84 ± 0.02	3.45 ± 0.02	4.05 ± 0.03
	Minimum peak value (N)		0.35 ± 0.25 × 10^−2^	0.35 ± 0.25 × 10^−2^	0.35 ± 0.25 × 10^−2^
